# *In Vitro* Evaluation of *Lavandula angustifolia* Essential Oil on Anti-*Toxoplasma* Activity

**DOI:** 10.3389/fcimb.2021.755715

**Published:** 2021-09-29

**Authors:** Na Yao, Jia-Kang He, Ming Pan, Zhao-Feng Hou, Jin-Jun Xu, Yi Yang, Jian-Ping Tao, Si-Yang Huang

**Affiliations:** ^1^ Institute of Comparative Medicine, College of Veterinary Medicine, Yangzhou University, Yangzhou, China; ^2^ Jiangsu Co-Innovation Center for Prevention and Control of Important Animal Infectious Diseases and Zoonosis, Yangzhou University, Yangzhou, China; ^3^ Jiangsu Key Laboratory of Zoonosis, Yangzhou University, Yangzhou, China; ^4^ College of Animal Science and Technology, Guangxi University, Nanning, China; ^5^ College of Animal Sciences, Zhejiang Provincial Key Laboratory of Preventive Veterinary Medicine, Institute of Preventive Veterinary Medicine, Zhejiang University, Hangzhou, China; ^6^ Joint International Research Laboratory of Agriculture and Agri-Product Safety, The Ministry of Education of China, Yangzhou University, Yangzhou, China

**Keywords:** *Toxoplasma gondii*, natural medicine, *Lavandula angustifolia* essential oil, *in vitro*, treatment

## Abstract

The current methods of treating toxoplasmosis have a number of side effects, and these therapies are only effective against the acute stage of the disease. Thus, development of new low toxicity and efficient anti-*Toxoplasma* drugs is extremely important. Natural products are important sources for screening new drugs; among them, essential oils (EOs) have efficacy in anti-bacterial, anti-inflammatory, anti-insect, and other aspects. In this study, 16 EOs were screened for their anti-*T. gondii* activity. *Lavandula angustifolia* essential oil (*La* EO)was found to have an anti-parasitic effect on *T. gondii*. The cytotoxicity of *La* EO was firstly evaluated using the MTT assay on human foreskin fibroblast (HFF) cells, and then the anti-*T. gondii* activity was evaluated by plaque assay. Finally, the invasion experiment and electron microscope observation were used to study the mechanism of *La* EO in anti-toxoplasma activity. The results indicated that the CC_50_ of *La* EO was 4.48 mg/ml and that *La* EO had activity against *T. gondii* and the inhibition was in a dose-dependent manner under safe concentrations. *La* EO was able to reduce *T. gondii* invasion, which may be due to its detrimental effect on changes of the morphology of tachyzoites. These findings indicated that *La* EO could be a potential drug for treating toxoplasmosis.

## Introduction

*Toxoplasma gondii* is a zoonotic parasite found worldwide, which can infect almost all warm-blooded animals and human beings (Chemoh et al., 2013). It can cause severe or even a fatal outcome in immunocompromised individuals, such as organ transplant patients and AIDS patients. Pregnant women who are primarily infected during pregnancy can develop neonatal malformations, miscarriage, chorioretinitis, blindness, intellectual disability, and hydrocephalus in the infected fetus. *T. gondii* propagates sexually in the definitive host cat and excretes infectious oocysts through feces (Martorelli Di Genova et al., 2019). In addition, *T. gondii* can reproduce without a definitive host because of its special ability of asexual reproduction. All infectious forms (tachyzoites, cysts, and oocysts) can be transmitted through the food chain (Hussain et al., 2017). Its reproductive patterns, routes of transmission, and resistance to the outside environment make it widely distributed.

Controlling toxoplasmosis has been a great challenge because no vaccine is currently available. Nowadays, drugs are widely used to control this disease; sulfonamides are the gold treatment of toxoplasmosis in clinic, especially in combination with pyrimidine (Wei et al., 2015). Although the effect is quite good, the side effects are quite serious, such as myelosuppression and teratogenic problems in early pregnancy (Schmidt et al., 2006). Many people have to give up treatment due to the side effects. The fundamental disadvantage of this combination, even if adverse reactions are not considered, is that it only solves the problem of acute infection, but has no effect on the underlying chronic infection (Mirzaalizadeh et al., 2018). Moreover, the emergence of drug-resistant strains has exacerbated this dilemma.

The difficulty of treating toxoplasmosis has motivated the search for new effective and less toxic anti-*T. gondii* drugs. Natural products have always been an important source of drug discovery and improvement (Newman and Cragg, 2012; Yuan et al., 2017). Most plants, such as those in the large *Lamiaceae* family, are known to be rich in a variety of aromatic oils, many of which have been studied for medicinal purposes (Waller et al., 2017; Bekut et al., 2018; Uritu et al., 2018). After being treated with 200 μg/mL *Lavandula angustifolia* (*La*) EO for 24 hours, all the *Schistosoma japonicum* were killed completely (Mantovani et al., 2013). The accumulation of excessive amyloid beta (Aβ) plague in the hippocampus can cause cognitive impairment, while the aqueous extract of *La* can inhibit amyloid beta (Aβ) accumulation to some extent and has strong free radical scavenging activity, thus improving impaired memory and learning ability (Soheili et al., 2019). *Mentha pulegium* essential oil showed significant anti-*Bacillus subtilis* and anti-*Proteus mirabilis* activity, while *Rosmarinus officinalis* essential oil had an inhibitory effect on *Listeria* monocytogenes, *Bacillus subtilis*, *Escherichia coli*, and *Leishmania spp* (Bakri et al., 2017). In addition, they all have significant antioxidant capacity. *Scutellaria baicalensis* is one of the important ingredients of proprietary Chinese medicine, and its extract has a series of biological functions such as antiviral, antibacterial, liver protection, and so on (Wang et al., 2018). Searching for antiparasitic drugs from natural sources has increased in recent years, and EOs continue to be a major source of biologically active new drugs. Therefore, in this study, a plant extract, *La* EO, was selected to evaluate the *in vitro* inhibitory effect on *T. gondii* and provide a basis for the development of drugs for the treatment of toxoplasmosis.

## Materials And Methods

### Culture of Cells and Parasites

Human foreskin fibroblast (HFF) cells were cultured in Dulbecco’s Modified Eagle Medium (DMEM, Gibco™, USA), supplemented with 100 IU/mL penicillin and 100 μg/mL streptomycin (Solarbio, Beijing, China), along with 10% heat-inactivated fetal bovine serum (FBS, Gibco^®^, USA). The experimental strain of *T. gondii*, GFP-RH, was maintained in HFF cells with 2% heat-inactivated FBS at 37°C and 5% CO_2_. To isolate the tachyzoites, heavily infected cells were scraped, and the parasites were released by passing the cells through a 27-gauge needle 3–5 times, and were centrifuged at 3,500 rmp for 10 min to purify tachyzoites. The final centrifugal precipitates were suspended with PBS and then counted using a hemocytometer.

### Essential Oils

The 16 EOs used in this experiment were provided by Guangxi University and dissolved in dimethyl sulfoxide (DMSO) in a 1:1 ratio. The solutions were then diluted with DMEM, such that the final concentration of DMSO in the samples used in the experiment was lower than 1.56% v/v. The species number of *Lavandula angustifolia* used in this study is GXCM 2019032.

### Cytotoxicity Tests

HFF cells (1× 10^5^ cells/well) were cultured in 96-well plates at 37°C and 5% CO_2_ for 24 h, then the cells were treated with different concentrations of EOs for 24 h. A 1.56% solution of DMSO in DMEM and DMEM containing 10% FBS and 0.01% penicillin-streptomycin was used as the vehicle control. The HFF cells’ viability were measured by the MTT (3-[4,5-methylthiazol-2-yl]-2,5-diphenyltetrazolium bromide) colorimetric method according to (Costa et al., 2018). 20 μL of MTT solution (5 mg/mL) was added to each well and allowed to incubate at 37°C with 5% CO2 for 3 h and then 200 μL of DMSO was added to dissolve the formazan crystals. Absorbance was measured at 490 nm using an iMark™ Microplate Absorbance Reader (BioRad, Hercules, CA, USA) and the 50% cytotoxic concentrations (CC_50_) were calculated using Graph Pad Prism 8.0. The cytotoxicity experiment was performed in triplicate, using three separate plates.

### Effect of EOs in *T. gondii* Plaque Assay

In order to make a preliminary identification of the anti-*T. gondii* ability of Eos, 100 tachyzoites of the GFP-RH strain were used to infect HFF monolayers in 6-well plates in DMEM with 2% FBS at 37°C and 5% CO2. 4 hours later, HFF cells were treated with safe concentrations of two different doses of EOs. The non-infected and untreated cells were used as a blank control. HFF cells were washed wish PBS 3 times after 6 or 7 days of culture. The washed product was fixed with methanol for 10 min and stained with 0.1% crystal violet for 30 min. After washing with PBS three times and drying naturally, the plaque formed by tachyzoite infection could be seen and photographed under microscope as previously mentioned (Bai et al., 2018).

### Effects of *La* EO on *T. gondii* Infections *In Vitro*

HFF cells were incubated in 24-well plates with 10% FBS in DMEM for 48 h at 37°C in an atmosphere containing 5% CO_2_. Then the medium was replaced by DMEM with 2% FBS and 10^4^ freshly released tachyzoites of the GFP-RH strain were added to each well. After 4 h, the extracellular parasites were removed and fresh medium containing either different concentrations of *La* EO (6.67mg/ml, 3.34mg/ml, 1.67mg/ml, 0.83mg/ml, 0.42mg/ml), 1.56% DMSO (vehicle control), or 10μg/ml SMZ (positive control) was added to each well. 32 hours later, fluorescence microscope was used to observe and photograph the growth of GFP-RH, and the growth of GFP-RH was statistically analyzed by Image-Pro-Express.

### Effect of La EO on the Invasion of *T. gondii*

The invasion experiments were performed according to Augusto et al (Augusto et al., 2018). A 6-well plate of HFF cells was prepared, and 3 ml of 2% FBS in DMEM medium was added to each well. 10^4^ GFP-RH and 1.67mg/ml *La* EO were added simultaneously to the wells, allowing the tachyzoites to invade host cells for 20 min, 40 min, or 60 min, respectively. The supernatant was gently absorbed and the cells were fixed with methanol for 10 min, and then washed three times with PBS. After this, 5% BSA/PBS solution was added and blocked for 1 h, then gently washed three times with PBS. Mouse anti-*Toxoplasma* SAG1 monoclonal antibodies (mAb), diluted (1:1000) with a 1% BSA/PBS solution, were added to each well, and incubated at room temperature for 2 h. Then, goat anti-mouse IgG H&L(FITC) secondary antibodies, diluted (1:1000) in 1% BSA/PBS, were added to 6-well plates and incubated at room temperature for 2 h. After washing thrice with PBS, 300 μL of 0.2% Triton X-100 was added, and the mixture was left for 30 min. Cells were then gently washed three times with PBS, and 300 μL of a 5% BSA/PBS solution was added dropwise for a second blocking. The antibodies were added as per the procedure described earlier, this time using goat anti-mouse IgG H&L (Alexa Fluor ^®^ 568) (ab175473) instead of the goat anti-mouse IgG H&L(FITC). Finally, 300 μL of 30% glycerol was added to each well. Five visual fields were randomly selected for observation under the × objective of the fluorescence microscope and the parasites in each field were counted. Three repetitions were performed to increase the accuracy of the experiment.

Tachyzoites that were unable to successfully invade the cells were dyed green by goat anti-mouse IgG H&L(FITC), while all tachyzoites in the field of vision (including the non-invading and successfully invading ones) were stained red by goat anti-mouse IgG H&L (Alexa Fluor ^®^ 568)(ab175473). The difference between the tachyzoites of the two colors is termed as the absolute invasion number of tachyzoites. The ratio of the invasion number to the total number of tachyzoites is termed as the invasion rate of tachyzoites.

### Scanning Electron Microscopy Analysis

In order to observe the ultrastructure of the surface of tachyzoites, 10^3^ purified tachyzoites were added to each tube, and then treated with1.67mg/ml *La* EO and1.56% DMSO and incubated at 37°C for 8 h respectively. The sample was washed twice with PBS immediately, and the precipitate obtained by centrifugation was fixed overnight with 2.5% glutaraldehyde at room temperature. After gradient dehydration of 30%, 50%, 70%, 80%, 90%, 95%, and 100% ethanol, the critical point drying was carried out. Gold was used as the coating material, and the surface of the sample was sprayed with gold and then observed by scanning electron microscopy.

### Statistical Analysis

The Prism 8.0 software was used to analyze all the data. The antiparasitic activity of *La* EO was analyzed by an unpaired *t*-test, while the invasion experimental data are processed by multiple *t-*test, to compare the results of the test groups and those of the control group (**P < 0.01, ***P < 0.001).

## Results

### Cytotoxicity of EOs

**Figure 1:**
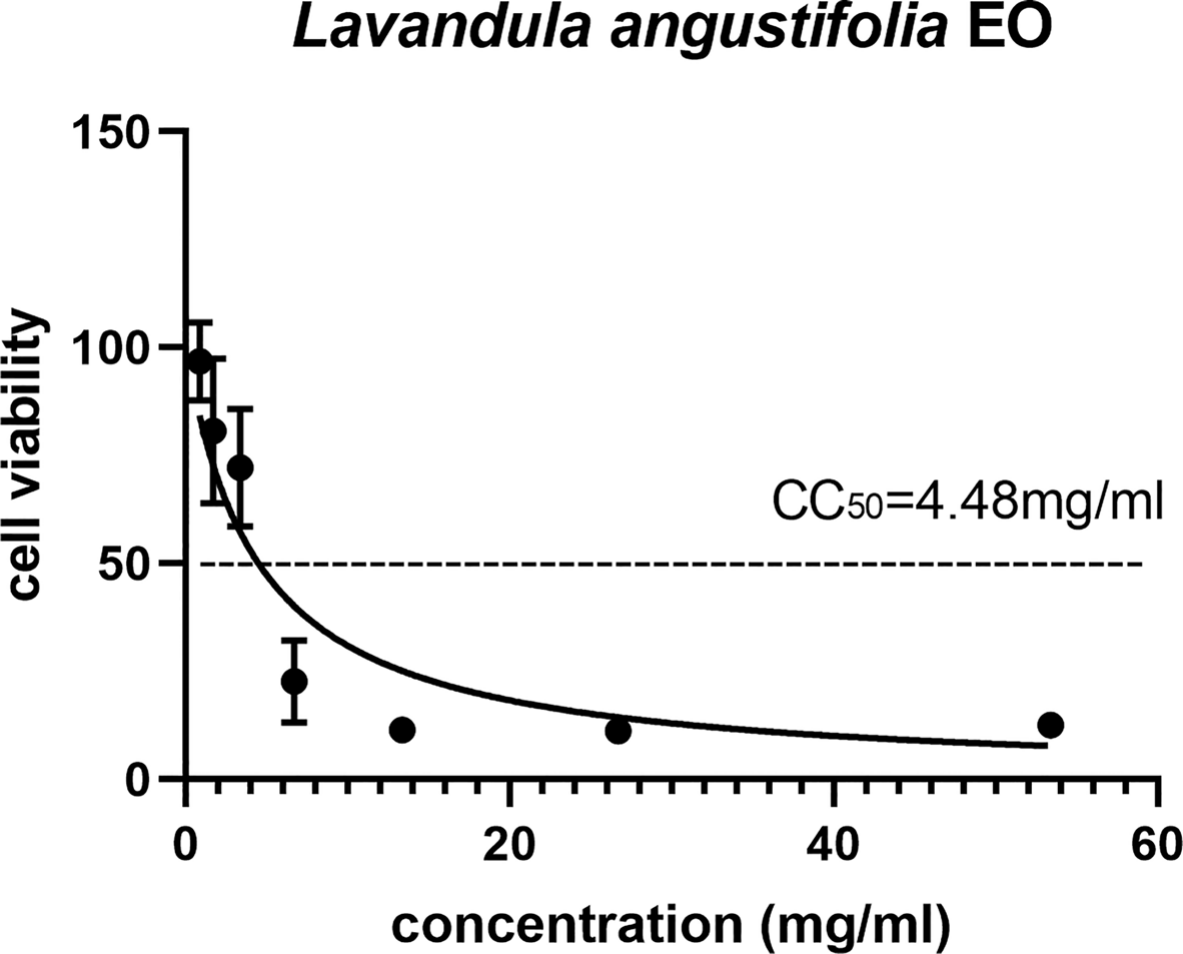
The 50% cytotoxic concentrations (CC50) of *La* EO. Cytotoxicity of La EO on HFF cells. Different concentrations of La EO were treated on HFF cells for 24 h and then Cytotoxicity was evaluated using MTT Assay. All data are presented with error bars and the experiments were performed in triplicate.

The cytotoxic potential of EOs on the HFF cell needed to be confirmed before further study. Among 16 EOs, 11 of them showed serious cell cytotoxicity, and only five of them had less cytotoxicity and could be further studied (**Table S1**); the CC_50_ of *La* EO was 4.48mg/ml, as shown in **Figure 1**.

### Antiparasitic Activity of *La* EO *In Vitro*

**Figure 2:**
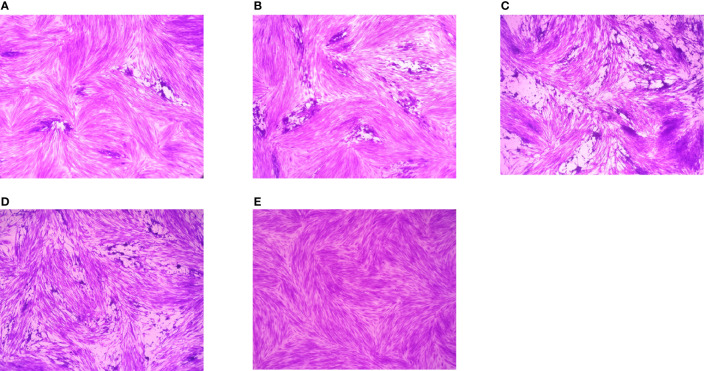
Plaque test for preliminary detection of anti-*T. gondii* activity. Images of *T. gondii* plaque under different concentrations of *La* EO. **(A)** HFF cells were infected by *T. gondii* and treated with 3.34mg/ml *La* EO; **(B)** HFF cells were infected by *T. gondii* and treated with 0.83mg/ml *La* EO; **(C)** HFF cells were infected by *T. gondii* and untreated; **(D)** HFF cells were infected by *T. gondii* and treated with DMSO **(E)** HFF cells were not infected and treated.

**Figure 3:**
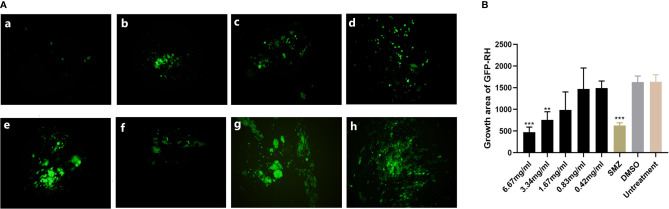
Anti-*T. gondii* activity of *La* EO evaluated by intracellular growth assay. **(A)** Fluorescence area indicates the growth of *T. gondii* during different treatment. (a–e) different concentrations of *La* EO, (a) 6.67mg/ml; (b) 3.34mg/ml; (c) 1.67mg/ml; (d) 0.83mg/ml; (e) 0.42mg/ml; (f) SMZ(10μg/ml); (g) DMSO; (h) no treatment. **(B)** Data analysis based on fluorescence area of RH-GFP. Each bar represents the mean ± SD of three wells per group. **P < 0.01, ***P < 0.001 compared with untreated group. All data are presented as with error bars and the experiments were performed in triplicate.

Preliminary plaque assay was used to screen the anti-*T. gondii* activity of EOs. Only *La* EO has anti-*T. gondii* activity (data not shown). From **Figure 2**, we can see that the plaques were smaller and fewer after being treated with two different concentrations of *La* EO, compared to those in the DMSO-treated and untreated groups. *La* EO has anti-*T. gondii* activity under these two safe concentrations. To conform the anti-*T. gondii* activity of *La* EO, gradient concentrations *La* EO were used to treat *T. gondii* infection, and the results showed that the growth of RH could be inhibited within the safe concentrations of *La* EO in a dose-dependent manner (**Figure 3**). **Figure 3** showed the growth of *T. gondii* was significantly reduced at 6.67mg/ml *La* EO treatment (474.7vs1636; 474.7vs1629, P<0.001) when compared to the untreated and 1.56%DMSO treated groups. There was also a significant difference between the 3.34mg/ml *La* EO treatment and control groups (756.3vs1636, 756.3vs1629, P <0.01), which indicated that the inhibition in 3.34mg/ml group is also very good, although the effect was not as good as that in SMZ group (756.3vs 627.3, P(0.0035)>P(0.0005).

### Effect of *La* EO on the Invasion of *T. gondii*

**Figure 4:**
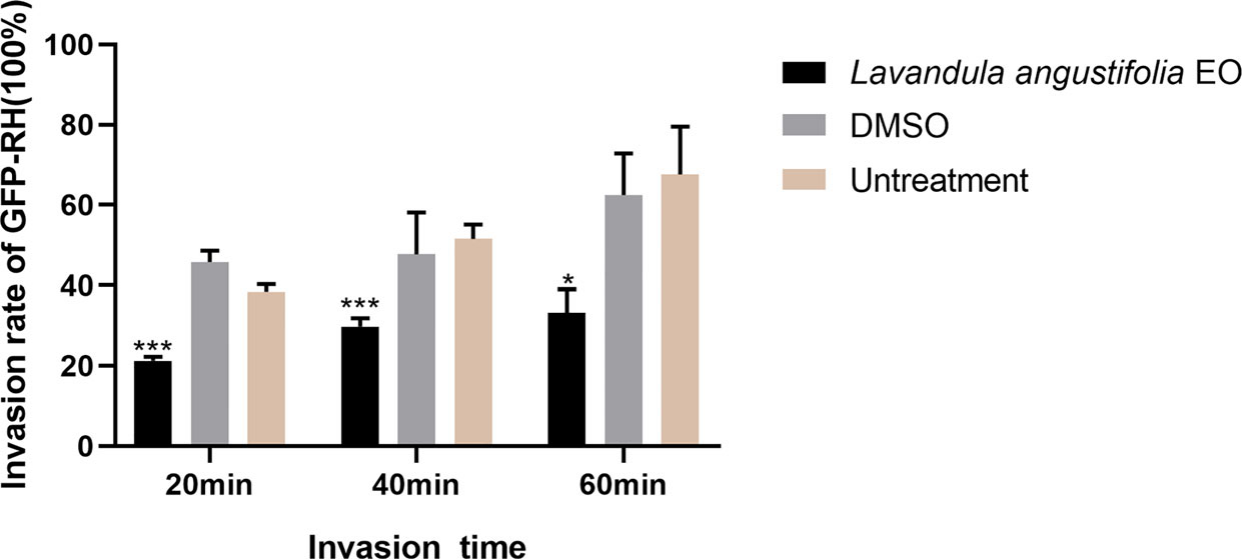
Effect of *La* EO on the invasion of *T. gondiii.* Statistics of *T. gondii* invasion rate using two immunofluorescent dyes after being treated with *La* EO for 20, 40, and 60 minutes, respectively. *P < 0.05, ***P < 0.001 compared with untreated group. All data are presented as with error bars and the experiments were performed in triplicate.

As shown in **Figure 4**, in the 3.34mg/ml *La* EO treatment group, the *T. gondii* invasion rates at 20 min, 40 min, and 60 min post-infection were found to be 21.3%, 29.77%, and 39.17%, respectively. For the untreated groups, the invasion was 38.50%, 51.51%, and 67.64%, respectively. It clearly indicated that *La* EO could inhibit the invasion of *T. gondii*, especially in 20- and 40-minutes groups (P <0.001). No change in the invasion rate of *T. gondii* was observed in any group treated with DMSO, across all experiments.

### Electron Microscopy Analysis

**Figure 5:**
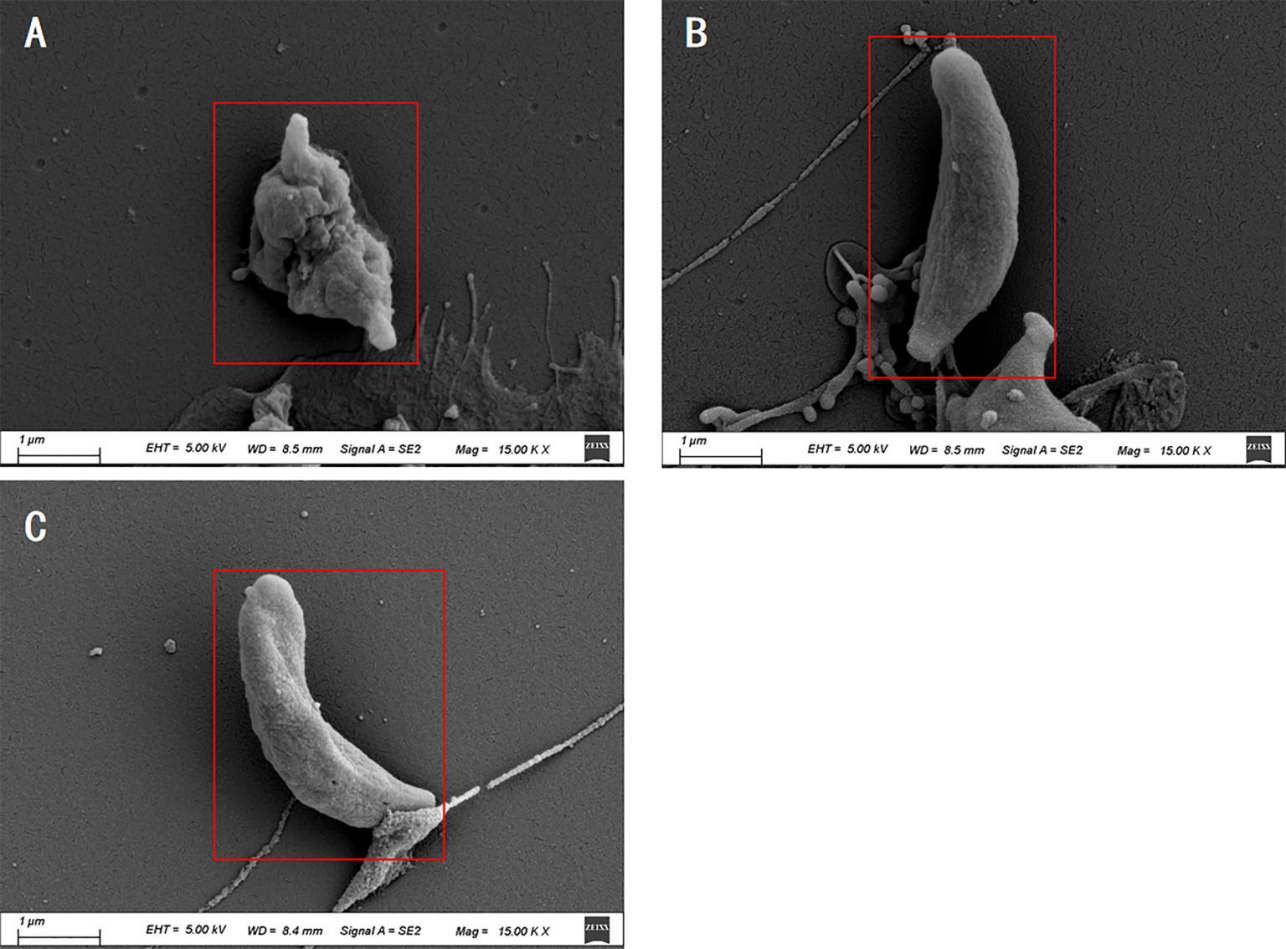
Scanning electron microscopy assay. *T.gondii* were treated with 3.34mg/mL *La* EO **(A)**, DMSO **(B)** or untreated **(C)**. After being treated by *La* EO, the tachyzoites became rough, wrinkled, and sunken compared with untreated tachyzoites, Scale bars: 1μm. The experiments were performed in triplicate.

The SEM results showed that tachyzoites were seriously deformed and shrunk after being treated by *La* EO and no longer maintain the crescent shape (**Figure 5A**) compared to no treatent group (**Figure 5C**) and DMSO-treated group (**Figure 5B**). *La* EO greatly changed the morphology and structure of tachyzoites, which seriously affected the movement ability and inhibited the invasion.

## Discussion

*T. gondii* has attained global attention due to its socioeconomic impacts and public health safety hazards, while the therapeutic drugs still have various limitations, such as side effects and drug resistance. Research into new anti-*toxoplasma* drugs is still urgent and important. Compared to chemical drugs, the natural drug resources are more abundant, therefore, exploring new drugs from natural products is worthy of consideration and, in fact, this research direction has a strong foundation in reality. For example, vanillin isolated from the pods of tropical plants can significantly improve the survival rate of Swiss-Webster albino infected with *T. gondii* ME49 (Oliveira et al., 2014). Pyrimethamine is the standard treatment drug of *T. gondii*, while the anti-*T. gondii* therapy of eucalyptus extract was superior to that of pyrimethamine in mouse survival rate and cell safety (Mirzaalizadeh et al., 2018). Due to these findings, we focused on *Lavandula angustifolia* from a potential family of Labiatae and tried to find drugs that have the anti-*T. gondii* activity.

According to the *in vitro* results, *La* EO showed higher CC50 than other EOs in HFF cells. Interestingly, *La* EO under concentration of 4.48mg/ml did not significantly reduce the viability in HFF cells. At the same time, *La* EO showed an anti-*T. gondii* activity in a dose-dependent manner in infected HFF cells. *La* EO significantly reduced the plaque sizes and numbers compared to the control groups; these results indicated that *La* EO inhibited the growth of *T. gondii* probably by inhibiting the invasion and intracellular proliferation. Lots of studies showed the effects of different herbal drugs against *T. gondii* infection *in vivo*. In this study we found that *La* EO has significant activity against *T. gondii*, although the main active ingredients were not clear. Our finding supported the idea that natural compounds and traditional herbal medicine are important candidates for searching for new anti-parasite drugs.

According to previous reports, the main active ingredients of lavender essential oil are linalool, terpineol, eucalyptus oil, lavender alcohol, and geraniol. (Białoń et al., 2019). Due to the presence of these ingredients, *La* EO is hydrophobic, so it easily penetrates the cell membrane (Ben Hsouna and Hamdi, 2012; Mantovani et al., 2013). Geraniol and terpineol, similar to octopamine (OA), can bind to specific G protein-coupled receptors, thereby affecting the concentration of cAMP and Ca2^+^, and then activate the corresponding kinases to exert their biological activities (Jankowska et al., 2017; Ebadollahi et al., 2020). It is well known that the kinase domain of CDPKs family can be directly regulated by calcium ion (Wernimont et al., 2010). CDPK1 is closely related to the adhesion and invasion of *T. gondii* (Johnson et al., 2012). Therefore, some components of *La* EO may affect the calcium concentration, and then inhibit the function of CDPK1, which causes the invasion to be significantly inhibited by *La* EO (**Figure 4**). Unfortunately, we did not find the OA-like receptors in *T. gondii*; better understanding this pathway will improve the development of new drugs. At the same time, cAMP is also closely related to the invasion of tachyzoites, which is also important for further drug development (Hartmann et al., 2013).

From the electron microscope results, we found that the surface of *T. gondii* tachyzoites became rough, wrinkled, and sunken after being treated by *La* EO compared to the control groups. The ultrastructure of *Toxoplasma* showed that La EO caused serious damage to the membrane of *T. gondii*. This chemical reaction results in a huge depression in the middle of the tachyzoite since the various components of the essential oil itself can damage the permeability of the cell membrane (Mantovani et al., 2013; Essid et al., 2017; Gucwa et al., 2018). The cAMP signal is generally believed to regulate mitochondrial initiation of apoptosis, and apoptosis can be promoted by maintaining a high level of intracellular cAMP (Valsecchi et al., 2013). As mentioned before, the cAMP levels can be increased by some components of EOs (Jankowska et al., 2017). It has been reported that some components of EO can disrupt ion channels, destroy the depolarization of mitochondrial membrane, cause electrolyte leakage, and make mitochondria permeable, thus causing *T. gondii* damage and death (Swamy et al., 2016). We hypothesized that *La* EO interfered with the normal metabolism of *T. gondii*, and the normal morphology cannot be maintained, so that the invasion is inhibited, and then the growth of *T. gondii* was inhibited. However, the accurate mechanism is still not clear and further studies need to be carried out.

## Conclusion

In summary, natural extracts are important sources for screening new drugs. *La* EO was found to have anti-*Toxoplasma* activity. The inhibitory effect may be due to the influence on the *T. gondii* shape, and then the invasion was inhibited. However, the specific mechanism of action from *La* EO on *T. gondii* is still unclear and warrants further studies.

## Data Availability Statement

The original contributions presented in the study are included in the article/**Supplementary Material**. Further inquiries can be directed to the corresponding author.

## Author Contributions

S-YH and NY conceived and designed the study. NY, J-KH, MP, and Z-FH performed the laboratory analyses. J-JX, YY, and J-PT analyzed the data. All authors critically appraised and interpreted the results. NY drafted the first version of the manuscript. All authors contributed to the article and approved the submitted version.

## Funding

The sample collection and some experiments were supported by the Outstanding Youth Foundation of Jiangsu Province of China (BK20190046), The China Postdoctoral Science Foundation (2020M671615), the Science and Technology Major Project of Zhejiang Province, China. (No. 2012C12009-2), and the Priority Academic Program Development of Jiangsu Higher Education Institutions (Veterinary Medicine).

## Conflict of Interest

The authors declare that the research was conducted in the absence of any commercial or financial relationships that could be construed as a potential conflict of interest.

## Publisher’s Note

All claims expressed in this article are solely those of the authors and do not necessarily represent those of their affiliated organizations, or those of the publisher, the editors and the reviewers. Any product that may be evaluated in this article, or claim that may be made by its manufacturer, is not guaranteed or endorsed by the publisher.
