# Supplementary material for: In Vitro Evaluation of Lavandula angustifolia Essential Oil on Anti-Toxoplasma Activity
Source: Front Cell Infect Microbiol. 2021 Sep 29;11:755715. doi: 10.3389/fcimb.2021.755715 (PMC8513107; doi:10.3389/fcimb.2021.755715)
Supplement: Supplementary file 1 [file Table_1.docx]

| **NO.** | **Essential Oils (EOs)** | **CC50** | **Cytotoxicity** | **Anti- T*oxoplasma*** |
| --- | --- | --- | --- | --- |
| 1 | *Lavandula angustifolia* EO | 4.48 mg/ml | - | + |
| 2 | *Rosmarinus officinalis* EO | 55.65 mg/ml | - | - |
| 3 | *Anthemis nobilis* EO | 16.67 mg/ml | - | - |
| 4 | *Curcuma kwangsiensis* EO | 2.897 mg/ml | + | - |
| 5 | *Illicium verum* EO | 10 mg/ml | - | - |
| 6 | *Cinnamomum cassia* EO | 657.8 μg/ml | + | - |
| 7 | *Syzygium aromaticum* EO | 469.8 μg/ml | + | - |
| 8 | Eugenol | 346.9 μg/ml l | + | - |
| 9 | *Vitex negundo* EO | 1.26 mg/ml | + | - |
| 10 | *Litsea cubeba* EO | 134.9 μg/ml | + | - |
| 11 | *Ocimum basilicum* EO | 43.93 μg/ml | + | - |
| 12 | *Rhododendron capitatum* EO | 2.77 mg/ml | + | - |
| 13 | *Mentha canadensis* EO | 15.01 mg/ml | - | - |
| 14 | *Salvia japonica* EO | 2.25 mg/ml | + | - |
| 15 | *Thymus mongolicus* EO | 1.26 mg/ml | + | - |
| 16 | *Cymbopogon citratus* EO | 2.74 mg/ml | + | - |

Table S1 The characteristic of cytotoxicity and anti-*Toxoplasma* of 16 EOs in this study.
